# Expression of vascular endothelial growth factor and basic fibroblast growth factor in acute rejection reaction following rat orthotopic liver transplantation

**DOI:** 10.3892/etm.2014.1779

**Published:** 2014-06-11

**Authors:** CHANGSONG ZHANG, GUANGSHUN YANG, DEWEN LU, YANG LING, GUIHUA CHEN, TIANBAO ZHOU

**Affiliations:** 1 Clinical Oncology Laboratory, Changzhou Tumor Hospital, Medical College of Soochow University, Changzhou, Jiangsu 213002, P.R. China; 2 Eastern Hepatobiliary Surgery Hospital, Second Military Medical University, Shanghai 200438, P.R. China; 3 The Affiliated Yinzhou Hospital, Ningbo University School of Medicine, Ningbo, Zhejiang 315040, P.R. China; 4 Hepatic Surgery Center, Third Affiliated Hospital, Sun Yat-Sen University, Guangzhou, Guangdong 510630, P.R. China; 5 Hepatobiliary Surgery Centre, Ningbo No. 2 Hospital, Ningbo, Zhejiang 315010, P.R. China

**Keywords:** orthotopic liver transplantation, acute rejection reaction, vascular endothelial growth factor, basic fibroblast growth factor

## Abstract

The aim of the present study was to investigate the expression levels of vascular endothelial growth factor (VEGF) and basic fibroblast growth factor (bFGF) in acute rejection reaction (ARR) following orthotopic liver transplantation in a rat model. Serum VEGF and bFGF levels were detected using ELISA, and their expression levels in liver and spleen tissues were determined using immunohistochemistry. The mRNA expression levels of VEGF and bFGF were detected by conducting a quantitative polymerase chain reaction during the ARR following orthotopic liver transplantation. The expression levels of VEGF and bFGF in the serum 3 days following liver transplantation were significantly higher compared with those in the other groups (1 and 7 days following transplantation; P<0.01). In addition, the numbers of cells in the liver tissue that were shown to be positive for the expression VEGF and bFGF using immunohistochemistry were significantly higher 3 days following transplantation than at the other time points (P<0.0001). Furthermore, the numbers of cells positive for VEGF and bFGF expression in the spleen detected 3 days following the transplantation surgery were also significantly higher compared with those at the other time points (P<0.01). VEGF and bFGF mRNA expression levels were also increased from 1 day following the surgery and reached a peak at day 3, prior to declining gradually and remaining at a relatively high level. VEGF and bFGF mRNA expression levels changed dynamically, by peaking and then declining, in ARR following orthotopic liver transplantation. These changes may have an important impact on angiogenesis and the inflammatory reaction, and the identification of these changes increases the current understanding of ARR following orthotopic liver transplantation.

## Introduction

Bile duct epithelial cells are considered to be the most important cells involved in acute rejection reaction (ARR) following orthotopic liver transplantation (1). However, recent studies have demonstrated that the interaction and time overlap between inflammatory cell infiltration and angiogenesis in the portal area are the primary causes of ARR following orthotopic liver transplantation (2). It has been reported that macrophages and lymphocytes stimulate angiogenesis via the release of angiogenic factors, including vascular endothelial growth factor (VEGF) and basic fibroblast growth factor (bFGF) (3,4). With respect to mechanism of action, VEGF is endothelial cell-specific; it stimulates endothelial proliferation, increases vascular permeability and changes the gene expression of endothelial cells. It is the most potent known vascular permeability agent (5,6). bFGF stimulates the proliferation of endothelial cells, smooth muscle cells and fibroblasts, as well as the formation of small arteries; however, bFGF does not increase vascular permeability (7).

The roles of VEGF and bFGF in mediating the interactions between and concurrent timing of inflammatory cell infiltration and angiogenesis in the portal area have yet to be elucidated. Therefore, this was investigated in the present study in order to further the understanding of ARR following orthotopic liver transplantation.

## Materials and methods

### Subjects and groups

The inbred line DA (RT1a) to LEW (RT11) rat orthotopic liver transplantation ARR model was established using the classic two-cuff technique as previously described by Kamada and Calne (8). The rats were purchased from the SLAC Laboratory Animal (Shanghai, China), and were housed in filter-capped polycarbonate cages and maintained under constant environmental conditions (average 22°C, humidity 50%). The rats were kept on a 12h/12h light-dark cycle and had unrestricted access to purified bottled drinking water and standard chow. A total of 48 rats were equally divided into a DA (RT1a)-LEW (RT11) ARR VEGF group and a DA (RT1a)-LEW (RT11) ARR bFGF group. The two groups were further divided into three subgroups by the number of days following transplantation (days 1, 3 and 7). A total of eight rats served as a control group without receiving any treatment. Histopathological classification of acute allograft reaction was performed according to the ‘Banff’ international criteria (9). Immunohistochemistry (IHC) studies were performed using the standard streptavidin-biotin-peroxidase complex method. In brief, tissue slides were deparaffinized and rehydrated. The slides were scanned using Motic Med 6.0 CMIAS (Motic China Group, Co., Ltd., Xiamen, China). The rejection activity index (RAI) was obtained by comprehensive analysis of VEGF and bFGF in liver acute rejection reaction. The study protocol was approved by Ethics Committee of the Second Military Medical University.

### Determination of VEGF and bFGF levels by ELISA

Serum VEGF and bFGF levels were detected by ELISA (double antibody sandwich ABC-ELISA method) using a VEGF-C and FGF-basic human ELISA kit (Invitrogen Corporation, Camarillo, CA, USA), by drawing 2 ml venous blood from the inferior vena cava at days 1, 3 and 7 following liver transplantation. The results were determined as follows: i) the absorbance (A) value at 450 nm was calculated by correcting for the blank value; ii) using the A value of the standard product, a standard curve was drawn on semi-logarithmic paper; iii) the VEGF and bFGF levels were then determined according to the A value of the sample using the standard curve.

### Detection of tissue VEGF and bFGF levels using immunohistochemistry

The expression levels of VEGF and bFGF in the transplanted liver and spleen tissues were assayed using immunohistochemistry. The rabbit anti-rat VEGF and bFGF immunoglobulin G1 monoclonal antibodies (Santa Cruz Biotechnology, Inc., Santa Cruz, CA, US) were used at ratios of 1:80 and 1:120, respectively. EnVision reagent (horseradish peroxidase/rabbit) was obtained from Dako (Glostrup, Denmark). The immunohistochemical results were analyzed quantitatively using a true color medical image analysis system (Motic Med 6.0 CMIAS; Motic China Group, Co., Ltd.). A positive result for VEGF was defined as the presence of yellow brown or dark brown particles in cytoplasm. First, the 10 most concentrated areas of positive cells were randomly selected (magnification, ×100), and then transferred to the CMIAS (magnification, ×400) to calculate the number of positive cells on the screen per mm^2^ by amplifying by a factor of 1.6. Known positive SNU-1 gastric cancer tissue (Shanghai Institutes for Biological Sciences, Shanghai, China) was used as the positive control, and phosphate-buffered solution was used instead of the primary antibody as the negative control.

### Determination of VEGF and bFGF mRNA expression in the liver tissue by quantitative polymerase chain reaction (qPCR)

VEGF and bFGF expression levels in the liver tissue were detected using a TRIzol kit (Shanghai Biological Engineering Technology Service Co., Ltd., Shanghai, China); reverse transcription (RT)-PCR (Takara, Shiga, Japan); two-step RT-PCR kit; and DL 1,000 DNA marker (Takara Biotechnology Co., Ltd., Dalian, China). The primers used for qPCR were as follows: VEGF forward, 5′-ACCTCACCAAAGCCAGCACA-3′ and reverse, 5′-GGC ATGGTGGTGACATGGTT-3′ (amplification product, 536 bp); bFGF forward, 5′-ACACGTCAAACTACAACT CCA-3′ and reverse, 5′-TCAGCTCTTAGCAGACATTGG-3′ (amplification product, 243 bp). qPCR was performed using the Mx3000P qPCR System (Stratagene, La Jolla, CA, USA). The cDNA was then used for qPCR in 20 μl SYBR Premix Ex Taq (Takara Biotechnology Co., Ltd). qPCR was performed under the following conditions: 5 min at 95°C, 40 cycles of 30 sec at 95°C, 30 sec at 60°C, and 1 min at 72°C. All results were normalized against β-actin amplification. CT values for triplicate reactions were averaged and relative expression was determined using the comparative CT method.

### Statistical analysis

All data are expressed as the mean ± standard deviation. Statistical analysis was performed using SPSS software, version 17.0 (SPSS, Inc., Chicago, IL, USA). Comparison of mean values between multiple groups was performed using a t-test. P<0.05 was considered to indicate a statistically significant difference.

## Results

### Serum VEGF and bFGF levels in ARR following rat orthotopic liver transplantation

**Figure 1:**
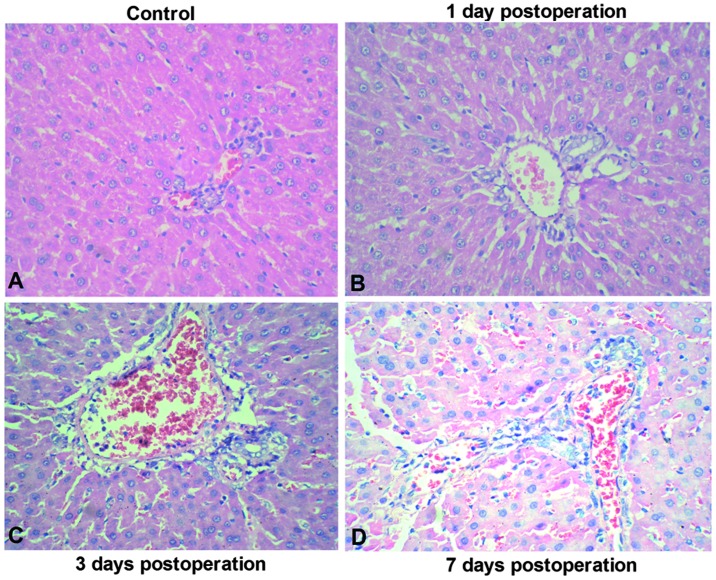
Representative hematoxylin and eosin staining in acute rejection reaction following rat orthotopic liver transplantation. (A) Normal rat liver tissue; (B) 1 day, (C) 3 days and (D) 7 days following rat orthotopic liver transplantation. (Magnification, ×20 objective lenses).

**Figure 2:**
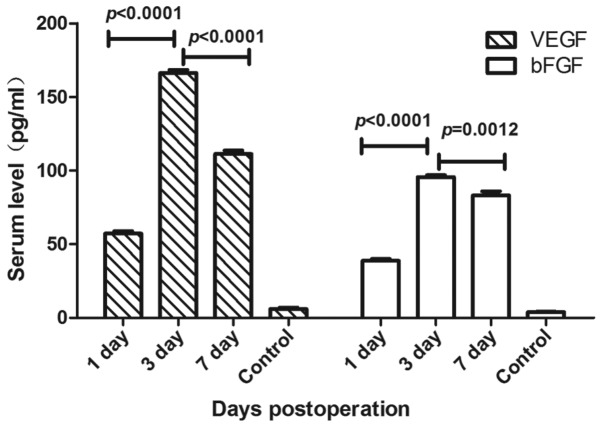
Serum VEGF and bFGF levels in acute rejection reaction following following rat orthotopic liver transplantation. The serum levels of VEGF and bFGF detected in the 3 day group were significantly higher compared with those in the other groups (P<0.001). VEGF, vascular endothelial growth factor; bFGF, basic fibroblast growth factor.

The rat orthotopic liver transplantation ARR model was established using the classic two-cuff technique. Acute allograft rejection was determined 1, 3 and 7 days following transplantation (Fig. 1). To accurately quantify serum VEGF and bFGF expression levels in ARR following rat orthotopic liver transplantation, ELISAs were performed 1, 3, and 7 days following transplantation. The serum VEGF and bFGF expression levels are shown in Fig. 2. Significant differences were observed in the VEGF level at day 3 compared with those on the other days (P<0.0001). It was found that the serum VEGF levels at day 3 were higher (mean, 166.30±2.16 pg/ml) than those on days 1 and 7 (mean, 57.16±1.61 and 111.0±2.43 pg/ml, respectively). Furthermore, serum bFGF levels were elevated at day 3 (mean, 95.64±1.26 pg/ml) compared with those on day 1 (mean, 38.74±1.35 pg/ml), and then decreased by day 7 (mean, 83.21±2.79 pg/ml). These results suggest that VEGF and bFGF levels may be critical for the development of ARR following rat orthotopic liver transplantation.

### Detection of VEGF and bFGF expression levels in the liver tissue using immunohistochemistry

Cell infiltration with large amounts of VEGF and bFGF expression was detected in the rats following transplantation, and there were significant differences in VEGF and bFGF expression between the three time points (P<0.0001).

**Figure 3:**
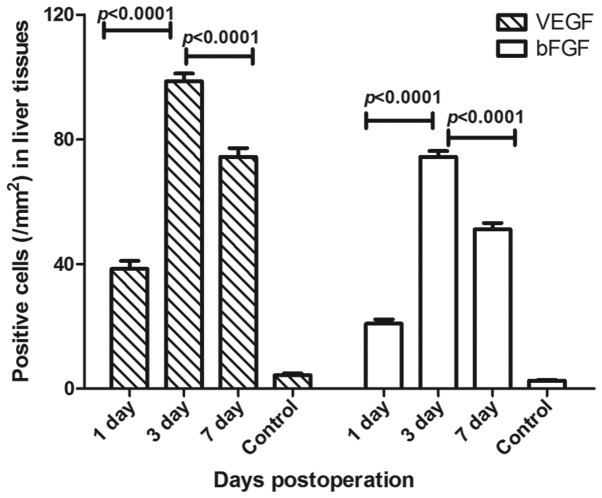
Intrahepatic expression of VEGF and bFGF in different subgroups of the orthotopic liver transplantation acute rejection reaction model. The numbers of cells positive for VEGF and bFGF in the 3 day group were significantly higher compared with those in the other groups, as detected by immunohistochemistry (P<0.0001). VEGF, vascular endothelial growth factor; bFGF, basic fibroblast growth factor.

The number of VEGF positive cells after 3 days was found to be higher (mean, 98.6±2.5/mm^2^) compared with the numbers on days 1 and 7 (mean, 38.4±2.6 and 74.3±2.8/mm^2^, respectively). Furthermore, the number of bFGF positive cells 3 days following transplantation was higher (mean, 74.4±1.9/mm^2^) compared with the numbers on days 1 and 7 (mean, 18.7±2.9 and 51.1±2.0/mm^2^, respectively; Fig. 3). However, only very low levels of VEGF expression in a small number of hepatocytes were observed between central vein endothelial cells and infiltrating cells, whereas bFGF expression was detected in this area.

### Detection of VEGF and bFGF expression in the spleen tissue using immunohistochemistry

**Figure 4:**
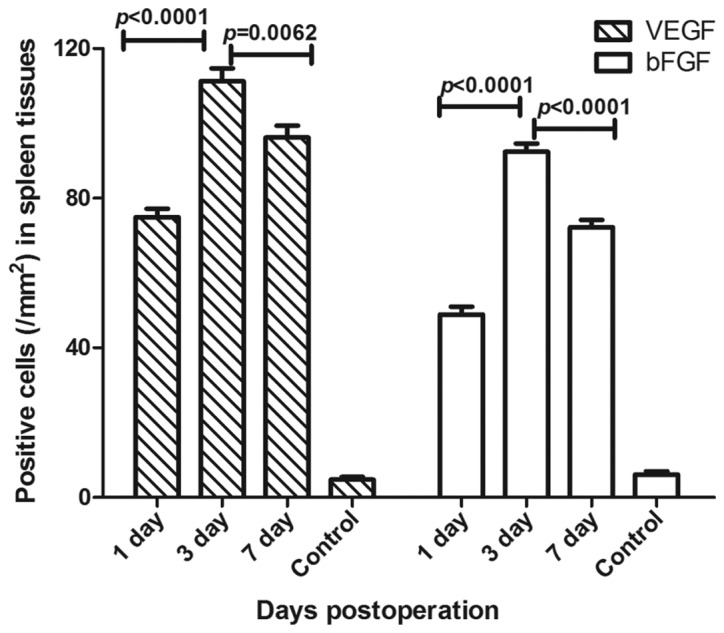
Intrasplenic expression of VEGF and bFGF in different subgroups of an orthotopic liver transplantation acute rejection reaction model detected using immunohistochemistry. The numbers of cells positive for VEGF and bFGF in the 3 day group were significantly higher compared with those in the other groups (P<0.01). VEGF, vascular endothelial growth factor; bFGF, basic fibroblast growth factor.

VEGF and bFGF expression levels were detected by immunohistochemistry in the spleen tissue, and there were significant differences between the three groups (P<0.01). It was found that the number of VEGF positive cells after 3 days was higher (mean, 111.3±3.4/mm^2^) compared with the numbers on days 1 and 7 (mean, 74.9±2.3 and 96.2±3.2/mm^2^, respectively). Furthermore, the number of bFGF positive cells after 3 days was also higher (mean, 92.4±2.2/mm^2^) compared with the numbers on days 1 and 7 (mean, 48.9±2.2 and 72.2±2.0/mm^2^, respectively; Fig. 4). VEGF was primarily expressed in the red pulp, and a small amount was expressed in the lymphatic sheath around the artery and the marginal area, with lymphocytes predominating and a small amount of macrophages. There was also a small amount of VEGF expression in the endothelial cells of the trabecular veins. bFGF expression was detected in the red pulp, and a small amount in the lymphatic sheath around the artery and the marginal area.

### VEGF and bFGF mRNA expression in the liver tissue

**Figure 5:**
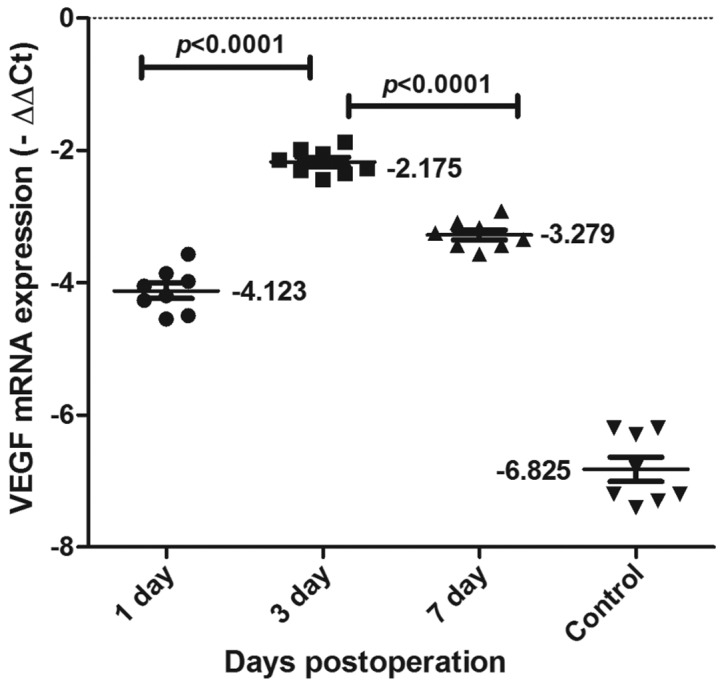
VEGF mRNA expression detected in the liver tissue by quantitative polymerase chain reaction. The level of VEGF mRNA expression was significantly higher in the 3 day group than in the other groups (P<0.0001). VEGF, vascular endothelial growth factor.

**Figure 6:**
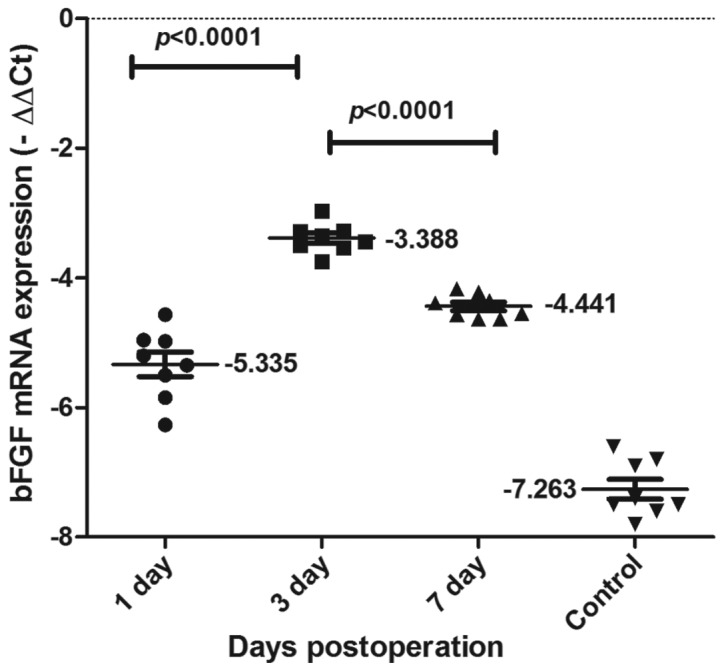
bFGF mRNA expression detected in the liver tissue by quantitative polymerase chain reaction. The level of bFGF mRNA expression was significantly higher in the 3 day group than in the other groups (P<0.0001). bFGF, basic fibroblast growth factor.

As shown in Figs. 5 and 6, VEGF and bFGF mRNA expression was detected using qPCR in each of the groups. VEGF and bFGF mRNA expression levels increased from 1 day following the surgery, reached a peak at day 3, and then declined gradually, although remaining at a relatively high level. VEGF and bFGF mRNA expression levels changed dynamically, peaking and then declining. Significant differences were observed between the three time-points (P<0.0001).

## Discussion

Bile duct epithelial cells are the most important cells involved in ARR following orthotopic liver transplantation. However, according to the three pathological changes summarized by Berman *et al* (10), the presentation of bile duct epithelial cell only reflects one aspect of ARR and does not explain the whole pathogenesis of ARR. This is supported by the fact that management of the bile duct alone does not solve the problem of ARR following orthotopic liver transplantation in clinical practice. Furthermore, bile duct epithelial cells also require normal expression of vascular endothelial cells so that they are able to obtain nutrients via microvessels and function normally (2).

Previous studies have shown that VEGF and bFGF are the primary growth factors that directly induce the division, proliferation and migration of endothelial cells and angiogenesis (11). In addition, immunoreactivity for VEGF has been found in the extracellular matrix of the portal tracts in normal and non-tumorous parts of liver, but not in the hepatocytes and bile duct epithelium (12). The results from the present study demonstrate that although the expression levels of VEGF and bFGF increased during the rejection process of liver transplantation, the bFGF expression level was lower; its expression was weaker, the scope of expression was wider, and the peak time was delayed compared with that of VEGF (Figs 5 and 6). This suggests that although VEGF and bFGF may mediate immunoinflammatory responses and angiogenesis in orthotopic liver transplantation, VEGF has a more important and specific role.

The exact role of VEGF in alloimmunity remains to be elucidated. Tambur *et al* (13) observed that VEGF was expressed in human allografted heart tissue, and that this expression was associated with ARR and chronic rejection. Furthermore, Conti *et al* (14) demonstrated that the inflammation-promoting effect of VEGF occurs mainly in the initial stages of the inflammatory cascade rather secondary to the T lymphocyte-mediated activation reaction, suggesting that VEGF is locally produced immediately following transplantation. This is consistent with the findings from the present study, that VEGF expression was enhanced on the first day following liver transplantation rejection. In addition, trauma, including the entry of platelets and white blood cell (WBC) supplements into the graft, further facilitates the expression of VEGF, and cytokines and chemokines that have important roles in the process of rejection. Early VEGF expression has been found to promote the repair of T lymphocytes and mononuclear cells (15). Furthermore, Fallsehr *et al* (16) observed that the action of VEGF on endothelial cells and macrophages activated nuclear factor κB, which subsequently induced the synthesis of inflammatory cytokines and chemokines.

A previous study demonstrated that intercellular adhesion molecule-1, vascular cell adhesion protein-1 and endothelial cell protein increased the adhesion of WBCs to the endothelial tissue and migration to the inflammatory area (17). In combination with the results from the present study, this suggests that VEGF production in liver tissues may be induced by anoxia or hypoxia, which is inevitable during transplantation, as well as via infiltration of neutrophils and macrophages into the graft.

Histologically, the spleen is made of white pulp, a marginal zone and red pulp. The marginal zone contains a greater number of T lymphocytes and macrophages and, therefore, the spleen is the first site at which antigens are captured and identified and an immunoresponse is triggered. The periarterial lymphatic sheath in the T-cell area of the spleen contains large amounts of T cells. In addition, the plenic cord of the red pulp contains large amounts of macrophages and T cells. It was found in the present study that VEGF was primarily expressed in the splenic marginal area containing T cells and macrophages, and rarely in the B-cell area. Microscopy demonstrated that the periarterial lymphatic sheath of the white pulp was thickened and the edge was widened, indicating that the cellular immunoresponse there was enhanced. Since the marginal area of the spleen is the first place where an immunoresponse is induced in the spleen, this suggests that VEGF expression in this area may promote immature dendritic cells to aggregate in the marginal area, where dendritic cells take up and process antigens that enter the spleen, and then directly submit them to T cells in the marginal area and activate them. It was also found in the present study that VEGF expression peaked 3 days following transplantation, which is consistent with the peak time of inflammatory cell infiltration during ARR, providing further support for the hypothesis that VEGF activates T cells during ARR following liver transplantation.

In conclusion, VEGF may be an important intermediary link between damage caused by physical, chemical and biological factors and subsequent immune injury due to the aggregation, activation and identification of lymphocytes and their effector cells in ARR following orthotopic liver transplantation. However, the exact mechanism underlying the action of VEGF and its applications require further investigation.
